# Adropin/Tirzepatide Combination Mitigates Cardiac Metabolic Aberrations in a Rat Model of Polycystic Ovarian Syndrome, Implicating the Role of the AKT/GSK3β/NF-κB/NLRP3 Pathway

**DOI:** 10.3390/ijms26010001

**Published:** 2024-12-24

**Authors:** Islam Ibrahim Hegab, Hemat El-sayed El-Horany, Rania Nagi Abd-Ellatif, Nahla Anas Nasef, Asmaa H. Okasha, Marwa Nagy Emam, Shereen Hassan, Walaa S. Elseady, Doaa A. Radwan, Rasha Osama ElEsawy, Yasser Mostafa Hafez, Maha Elsayed Hassan, Nouran Mostafa Mansour, Gamaleldien Elsayed Abdelkader, Mohamed H. Fouda, Amira M. Abd El Maged, Hanan M. Abdallah

**Affiliations:** 1 Physiology Department, Faculty of Medicine, Tanta University, Tanta 31527, Egypt; islam.hegab@med.tanta.edu.eg (I.I.H.); marwa.emam@med.tanta.edu.eg (M.N.E.); sheren.abdallh@med.tanta.edu.eg (S.H.);; 2 Bio-Physiology Department, Ibn Sina National College for Medical Studies, Jeddah 21442, Saudi Arabia; 3 Medical Biochemistry Department, Faculty of Medicine, Tanta University, Tanta 31527, Egypt; hemat.elhorany@med.tanta.edu.eg (H.E.-s.E.-H.); rania.emam@med.tanta.edu.eg (R.N.A.-E.); asmaa.hamdy@med.tanta.edu.eg (A.H.O.); 4 Department of Biochemistry, College of Medicine, Ha’il University, Hail 81158, Saudi Arabia; 5 Anatomy and Embryology Department, Faculty of Medicine, Tanta University, Tanta 31527, Egypt; walaa.elssaidi@med.tanta.edu.eg (W.S.E.); doaa.radwan@med.tanta.edu.eg (D.A.R.); 6 Pharmacology Department, Faculty of Medicine, Tanta University, Tanta 31527, Egypt; rasha.elesawy@med.tanta.edu.eg; 7 Internal Medicine Department, Faculty of Medicine, Tanta University, Tanta 31527, Egypt; yasser.hafez@med.tanta.edu.eg (Y.M.H.); maha.hassan@med.tanta.edu.eg (M.E.H.); 8 Cardiology Department, Faculty of Medicine, Tanta University, Tanta 31527, Egypt; dr.nouran@hotmail.co.uk; 9 Department of Restorative Dentistry and Basic Medical Sciences, Faculty of Dentistry, University of Petra, Amman 11196, Jordan; gamal.abdelkader@uop.edu.jo; 10 Clinical Pathology Department, Faculty of Medicine, Tanta University, Tanta 31527, Egypt; mohamed.fouda@med.tanta.edu.eg; 11 Pathology Department, Faculty of Medicine, Menoufia University, Shebin El Kom 32511, Egypt; amiramahrous85@gmail.com

**Keywords:** adropin, apoptosis, cardiovascular, inflammation, polycystic ovarian syndrome, tirzepatide

## Abstract

Polycystic ovarian syndrome (PCOS) is a multifaceted metabolic and hormonal disorder in females of reproductive age, frequently associated with cardiac disturbances. This research aimed to explore the protective potential of adropin and/or tirzepatide (Tirze) on cardiometabolic aberrations in the letrozole-induced PCOS model. Female Wistar non-pregnant rats were allotted into five groups: CON; PCOS; PCOS + adropin; PCOS + Tirze; and PCOS + adropin+ Tirze. The serum sex hormones, glucose, and lipid profiles were securitized. Cardiac phosphorylated levels of AKT(pAKT), glycogen synthase kinase-3 beta (pGSK-3β), NOD-like receptor family pyrin domain containing 3 (NLPR3), IL-1β and IL-18 were assayed. The cardiac redox status and endoplasmic reticulum stress (ER) parameters including relative glucose-regulated protein 78 (GRP78) and C/EBP homologous protein (CHOP) gene expressions were detected. Finally, the immunoreactivity of cardiac NF-κB, Bcl2, and BAX were assessed. Our results displayed that adropin and/or Tirze intervention successfully alleviated the PCOS-provoked cardiometabolic derangements with better results recorded for the combination treatment. The synergistic effect of adropin and Tirze is mostly mediated via activating the cardiac Akt, which dampens the GSK3β/NF-κB/NLRP3 signaling pathway, with a sequel of alleviating oxidative damage, inflammatory response, ER stress, and related apoptosis, making them alluring desirable therapeutic targets in PCOS-associated cardiac complications.

## 1. Introduction

Polycystic ovary syndrome is an intricate metabolic and endocrine disorder, clinically identified by the existence of two or more of the following manifestations: hyperandrogenemia, persistent anovulation, and polycystic ovaries [1]. Based on the NIH and Rotterdam diagnostic criteria, the PCOS prevalence was found to vary globally, with Middle Eastern populations having an estimated 8.9% and 11.9% prevalence, similar to global estimates of 5–9% and 10–20%, respectively [2].

The letrozole-induced PCOS model is broadly used, as it closely mirrors diverse features of human PCOS. It also persuades metabolic perpetuations such as dyslipidemia and insulin resistance (IR), which are relevant to PCOS-provoked metabolic disturbances [3].

The fundamental etiology of PCOS is not yet fully elucidated, despite how common it is. Substantial evidence implies that hyperandrogenism and IR are the pivotal drivers of its pathophysiology, in addition to influences of environmental factors, genetics, and epigenetics. PCOS has been significantly associated with the well-known traditional risk factors of cardiovascular disease (CVD), type II diabetes mellitus, hypertension, and metabolic syndrome, with its attendant cardiovascular complications, including stroke and coronary heart disease, recently referred to as PCOS distant complications, which has led to increased morbidity and mortality in women of reproductive age [4].

These serious cardiovascular events are preceded by a long period of subclinical pathological changes that require urgent attention for early characterization, which necessitate early intervention to reduce the future risk of CVD. However, studies reporting the association of PCOS with cardiac metabolic changes are largely lacking and the underlying mechanisms involved are still poorly understood.

Insulin resistance and hyperinsulinemia are the critical mediators of CVD in women with PCOS [5]. The cardiomyocyte is an insulin-targeted cell; insulin triggers phosphatidylinositol 3-kinase (PI3K)-Akt signaling and enhances the recruitment of glucose transporter 4 to the cell surface, facilitating the transport of glucose. A major downstream signaling molecule of Akt is glycogen synthase kinase-3 (GSK-3), a ubiquitously expressed serine/threonine kinase that has various cellular biological functions, including the regulation of metabolism, inflammation, and cell growth/death. GSK-3β is the predominant form in skeletal and cardiac muscles. Dysregulation of the Akt-GSK-3β signaling pathway is implicated in the cardiac pathogenic events associated with IR in different models, and it is considered an efficient therapeutic target for these conditions [6,7,8].

Adropin is a 76 amino acid circulating peptide, primarily expressed in the liver and brain, and modulates metabolic homeostasis in various tissues. Adropin was demonstrated to play an essential role in activating downstream insulin signaling, maintaining glucose homeostasis and lipid metabolism, and guarding against obesity-related hyperinsulinemia and hepatic steatosis [9,10,11,12]. More recently, adropin was reported to modulate cardiac energy metabolism in obese pre-diabetic mice downstream of AKT [13].

In the same context, type II diabetic patients reported lower levels of serum adropin than non-diabetic patients, which was correlated with coronary atherosclerosis angiographic severity [14]. Also, the serum adropin level was reported as a potential biomarker reflecting the status of cardiac damage and necrosis in coronary heart disease patients [15]. Based on these observations, adropin may confer a modulatory effect on cardiovascular pathogenesis associated with PCOS.

Tirzepatide is an innovative dual glucose-dependent insulinotropic polypeptide (GIP) and glucagon-like peptide-1 (GLP-1) receptor agonist approved for type II diabetes therapy, improving glycemic control to a greater extent than selective GLP-1 receptor agonists [16]. It was reported that type II diabetes patients’ treatment with Tirze resulted in the reduction in several inflammations, endothelial dysfunction, and cellular stress biomarkers associated with cardiovascular risk [17]. Moreover, favorable effects of Tirze on CVD risk factors were documented [18]. Therefore, it is plausible to speculate that it may have an impact on the progression of the cardiac pathogenic events in PCOS.

Taken together, this study was designed to explore the prospective protective impact of adropin and/or Tirze against cardiac pathophysiological events in experimentally induced PCOS in rats, highlighting the possible mechanisms that could underlie their mechanisms of action.

## 2. Results

### 2.1. Adropin and/or Tirze Ameliorated the PCOS-Induced Alterations in Blood Glucose, Serum Insulin, HOMA/IR, and Both Hormonal and Lipid Profiles

**Figure 1:**
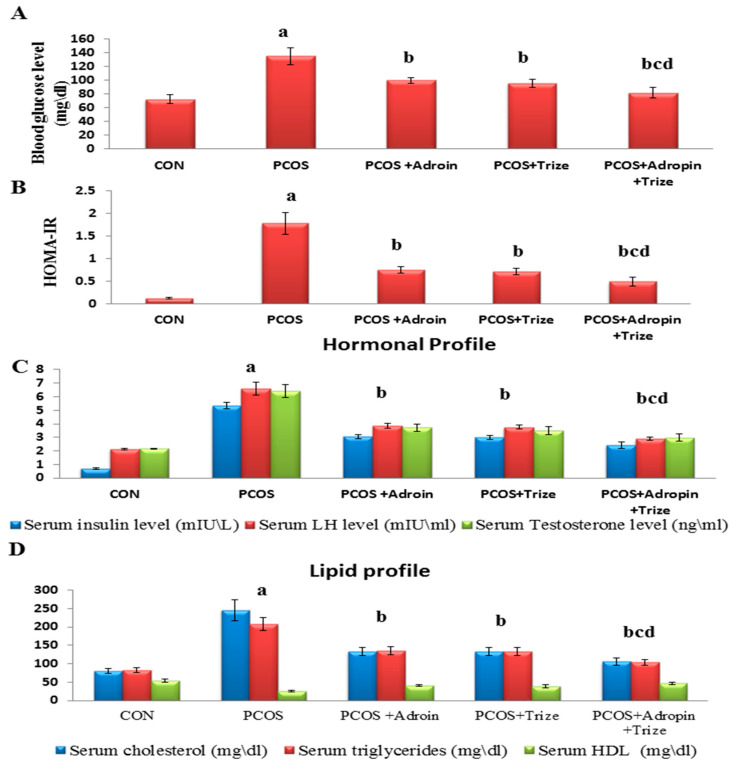
Impact of adropin and/or Tirze on blood glucose, serum insulin, HOMA/IR, and both hormonal and lipid profiles. (**A**) Blood glucose level (mg/dL), (**B**) HOMA-IR, (**C**) Hormonal profile and (**D**) Lipid profile. Data are represented as mean ± standard deviation. Statistical analysis was performed using one-way ANOVA with Tukey’s post hoc test, SPSS computer program. CON: control; Trize: tirzepatide; HOMA/IR: homeostasis model assessment of insulin resistance; LH: luteinizing hormone; HDL: High-density lipoprotein. ^a^ Significance versus CON group (*p* < 0.05), ^b^ Significance versus PCOS group (*p* < 0.05), ^c^ Significance versus PCOS + adropin group (*p* < 0.05) and ^d^ Significance versus PCOS + Tirze group (*p* < 0.05). n = 6 rats/each group.

As explicated in Figure 1 and relative to the CON group, the PCOS group demonstrated a substantial increment in blood glucose, serum insulin, and HOMA/IR levels concomitant with a remarkable elevation in the cholesterol and triglyceride levels, while HDL level was markedly lower. Notably, administration of either adropin or Tirze significantly reversed the overmentioned letrozole-induced changes in glucose and lipid profiles; meanwhile, the rats that received the adropin/Tirze combination scored the most favorable outcomes (Figure 1).

Oligoovulation, anovulation, and hyperandrogenism are the major criteria of PCOS. Hence, LH and testosterone serum levels were assayed as makers of steroidogenic function in the letrozole-induced PCOS rats. The PCOS group exhibited an obvious heightened LH and testosterone serum levels comparable to the CON group. In the meantime, their levels were substantially decreased in all treated PCOS groups, when compared with the untreated PCOS group. Remarkably, the adropin/Tirze combination group revealed a more pronounced regulatory effect on the hormonal profile (Figure 1).

### 2.2. Adropin and/or Tirze Intervention Reversed the PCOS-Induced Disturbance in the Cardiac AKT/GSK-3β Signaling Pathway

**Figure 2:**
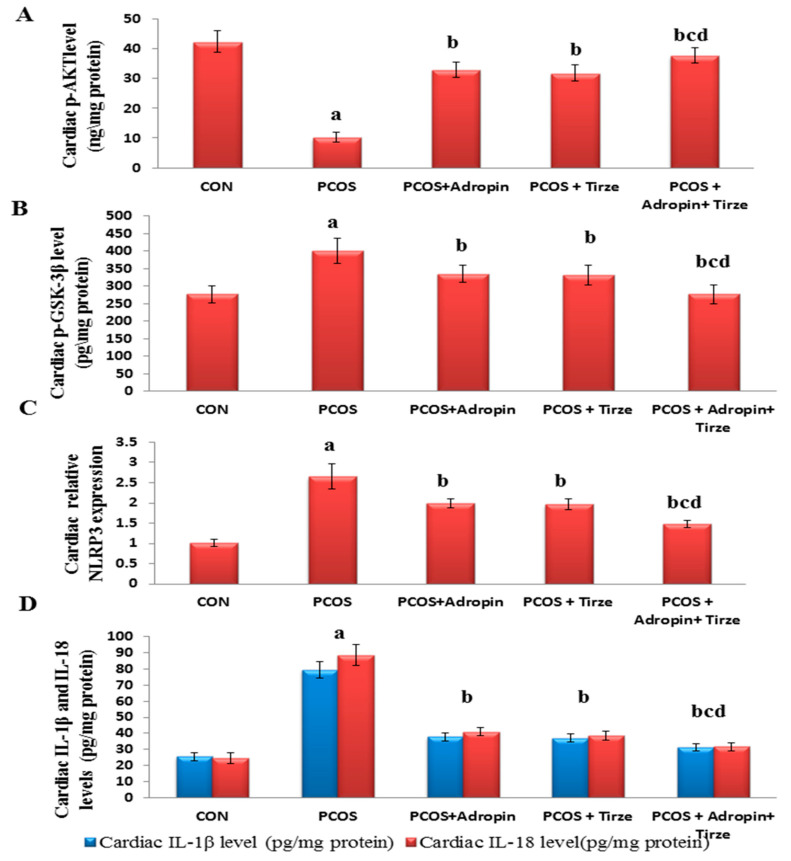
Impact of adropin and/or Tirze on cardiac levels of p-AKT, p-GSK-3β, IL-1β, and IL-18 and relative NLRP3 expression. (**A**); Cardiac AKT level (ng/mg protein), (**B**): Cardiac p-GSK-3β (pg/mg protein), (**C**): Cardiac relative NLRP3 expression and (**D**) Cardiac IL-1β and IL-18 levels (pg/mg protein). Data are represented as mean ± standard deviation. Statistical analysis was performed using one-way ANOVA with Tukey’s post hoc test, SPSS computer program. CON: control; Trize:tirzepatide; p-AKT: phosphorylated protein kinase B; p-GSK-3β: phosphorylated glycogen synthase kinase-3β; NLRP3: NOD-like receptor family pyrin domain containing 3; IL-1β: interleukin-1β; IL-18: interleukin-18. ^a^ Significance versus CON group (*p* < 0.05), ^b^ Significance versus PCOS group (*p* < 0.05), ^c^ Significance versus PCOS + adropin group (*p* < 0.05) and ^d^ Significance versus PCOS + Tirze group (*p* < 0.05). n = 6 rats/each group.

As dysregulation of the Akt/GSK-3β signaling pathway is implicated in IR-associated cardiac pathogenesis, we scrutinized the levels of their phosphorylated forms in cardiac tissue. The PCOS group unveiled a notable decline in the cardiac p-AKT level and an elevation in the pGSK-3β level when compared to the CON group. In parallel, the PCOS rats displayed profound elevation in the cardiac expression levels of NLRP3 along with IL-1β and IL-18 levels. Conversely, all treated PCOS groups were able to demonstrate an obvious recovery with the adropin/Tirze combination scoring a more pronounced reverse in these markers when compared to the use of each drug alone (Figure 2).

### 2.3. Impact of Adropin and/or Tirze on the Cardiac Redox State

**Figure 3:**
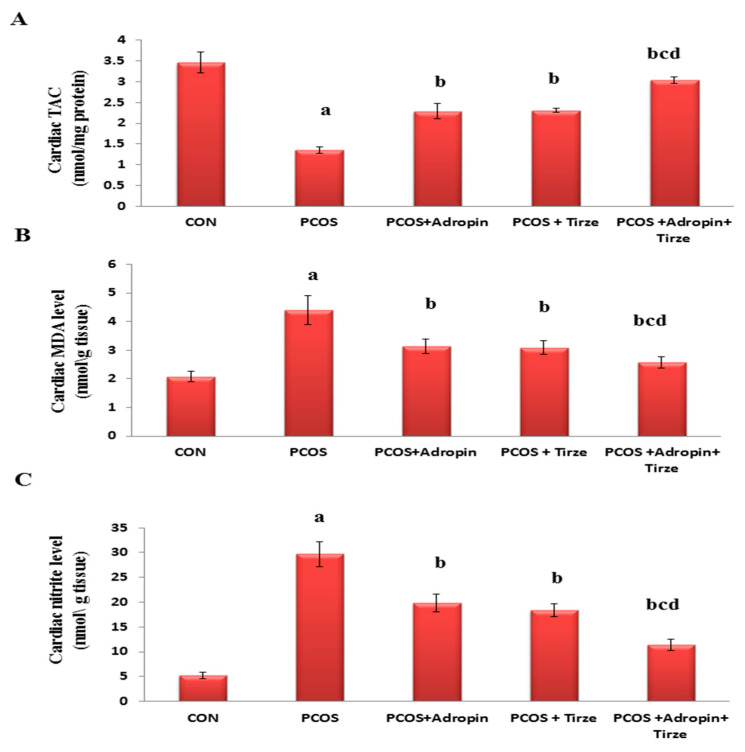
Impact of adropin and/or Tirze on cardiac TAC, MDA, and nitrite. (**A**) Cardia TAC (nmol/mg protein), (**B**) Cardiac MDA level (nmol/g tissue) and (**C**) Cardiac nitrite level (nmol/g tissue). Data are represented as mean ± standard deviation. Statistical analysis was performed using one-way ANOVA with Tukey’s post hoc test, SPSS computer program. CON: control; Trize: tirzepatide; TAC: total antioxidant capacity; MDA: Malondialdehyde. ^a^ Significance versus CON group (*p* < 0.05), ^b^ Significance versus PCOS group (*p* < 0.05), ^c^ Significance versus PCOS + adropin group (*p* < 0.05), and ^d^ Significance versus PCOS + Tirze group (*p* < 0.05). n = 6 rats/each group.

With regard to cardiac redox state parameters, the TAC was profoundly lower, whereas MDA and nitrite levels were substantially higher in the cardiac tissues of the PCOS rats relative to those of the CON ones. Interestingly, both adropin and Tirze demonstrated a significant recovery in the cardiac redox state parameters with the maximal response in favor of the adropin/Tirze combined intervention (Figure 3).

### 2.4. Impact of Adropin and/or Tirze on the Cardiac ER Stress Markers; GRP78 and CHOP and Apoptotic Marker Cleaved Caspase-3

**Figure 4:**
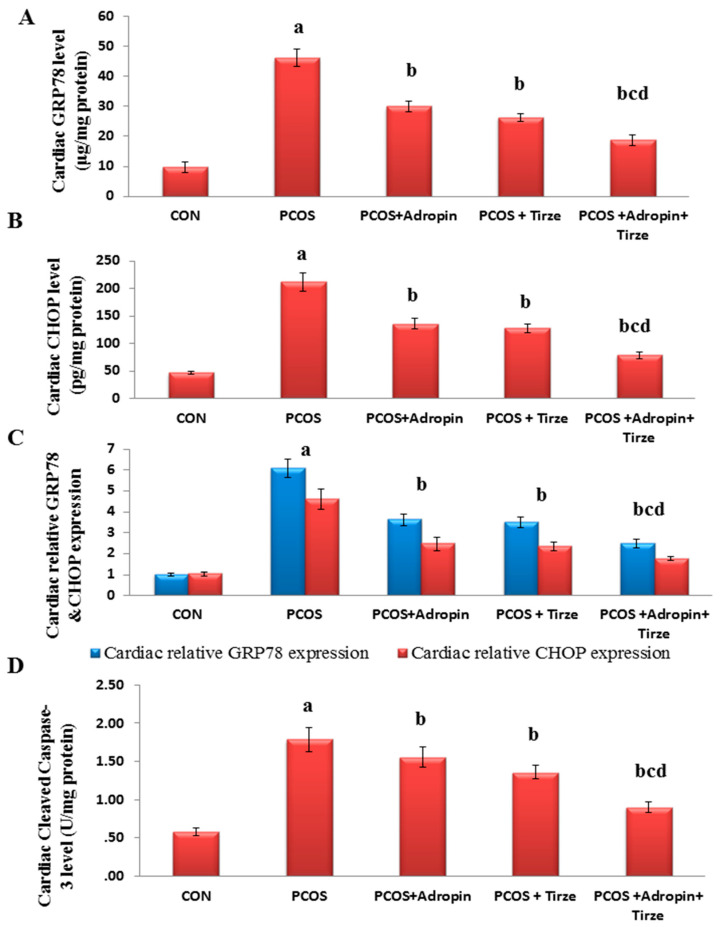
Impact of adropin and/or Tirze on cardiac levels of GRP78, CHOP, and cleaved caspase-3 and relative expression of GRP78 and CHOP. (**A**) Cardiac GRP78 level (μg/mg protein), (**B**) Cardiac CHOP level (pg/mg protein), (**C**) Cardiac relative GRP78 & CHOP expression and (**D**) Cardiac cleaved caspase-3 level (U/mg protein)Data are represented as mean ± standard deviation. Statistical analysis was performed using one-way ANOVA with Tukey’s post hoc test, SPSS computer program. CON: control; Trize: tirzepatide; GRP78: glucose-regulated protein 78; CHOP: C/EBP homologous protein. ^a^ Significance versus CON group (*p* < 0.05), ^b^ Significance versus PCOS group (*p* < 0.05), ^c^ Significance versus PCOS + adropin group (*p* < 0.05) and ^d^ Significance versus PCOS + Tirze group (*p* < 0.05). n = 6 rats/each group.

Based on the ER stress as a crucial inducer of apoptosis and cardiac injury, we assessed the cardiac levels of GRP78, CHOP, and cleaved caspase-3 and mRNA expression of GRP78 and CHOP. Our results scored noticeably elevated GRP78 and CHOP levels and mRNA expressions coupled with a significant increase in the level of cleaved caspase-3 in the PCOS rats relative to the CON rats. All treatments demonstrated a marked decline in these levels in contrast to the PCOS rats. Particularly, the Adropin/Tirze combination group revealed a more pronounced decline in the ER stress markers and cleaved caspase-3 level and ameliorated letrozole-induced changes in contrast to monotherapy (Figure 4).

### 2.5. Impact of Adropin and/or Tirze on Histological, Immunohistochemical, and Electron Microscopic Results

#### 2.5.1. Hematoxylin and Eosin Staining

**Figure 5:**
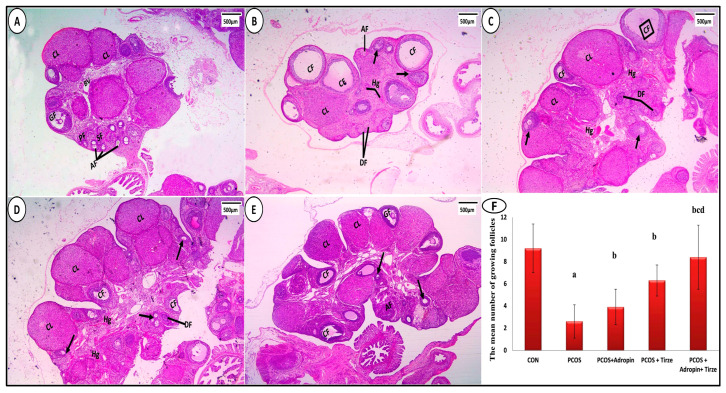
Sections of ovaries stained with H&E. (**A**) **CON Group** displaying normal ovarian architecture. The cortex reveals multiple primary follicles (PF), secondary follicles (SF), antral follicles (AF), Graafian follicles (GF), and average-sized corpora lutea (CL). Small blood vessels (BV) could be seen in the ovarian medium. (**B**) **PCOS group** showing multiple cystic follicles (CF), few growing follicles (arrows), one corpus luteum (CL), atretic follicles (AT), and degenerated follicles (DF). Hemorrhage (Hg) is seen between the follicles. (**C**) **The PCOS + Adropin group** showing a few cystic follicles (CF), many growing follicles (arrows), and corpora lutea (CL). However, degenerated follicles (DF) and hemorrhage (Hg) are noticeable. (**D**) **PCOS + Tirze Group** demonstrating an increased number of corpora lutea (CL), developing follicles (arrows), and fewer cystic follicles (CF). However, hemorrhage (Hg) and degeneration follicles (DF) are detected. (**E**) **PCOS + Adropin + Tirze Group** has several mature Graafian follicles (GF), developing follicles (arrows), and corpora lutea (CL). There are very few cystic (CF) and atretic follicles (AF). [Magnification ×40 scale bar = 500 µm]. (**F**) A column graph showing the mean number of growing follicles in the studied groups. ^a^ Significance versus CON group (*p* < 0.05), ^b^ Significance versus PCOS group (*p* < 0.05), ^c^ Significance versus PCOS + adropin group (*p* < 0.05) and ^d^ Significance versus PCOS + Tirze group (*p* < 0.05). n = 6 rats/each group.

**Figure 6:**
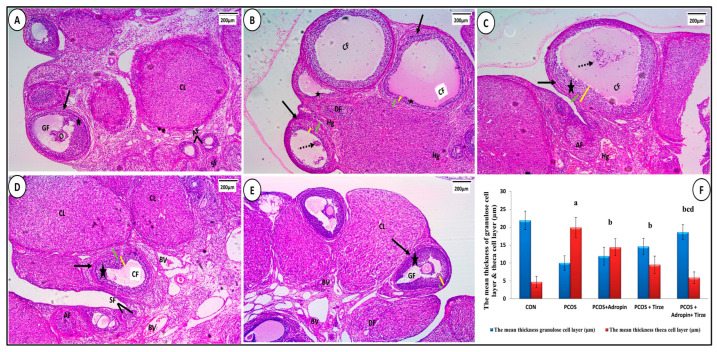
At a higher magnification. (**A**) **CON Group** showing a mature Graafian follicle (GF) with an oocyte (O) surrounded by a thick granulosa layer (star) and thin theca cell layer (arrow). Several secondary follicles (SF) and corpora lutea (CL) could be seen. (**B**) **PCOS Group** showing multiple cystic follicles (CF) that are lined by a thin granulosa layer (star) and a thick theca cell layer (arrows). Intraluminal desquamated granulosa cells (dotted arrow), degenerated corpus luteum (DF), and hemorrhage (Hg) between follicles are detected. (**C**) **PCOS + Adropin Group** depicting one cystic follicle (CF) with detached granulosa cells (dotted arrows) inside its lumen and bordered by theca (arrow) and partially thick granulosa (star) cell layers. Congested blood vessels (BV) and atretic follicles (AF) are notable. (**D**) **PCOS + Tirze Group** showing multiple corpora lutea (CL), secondary follicles (SF), and one cystic follicle (CF) surrounded by thick granulosa cell (star) and thick theca cell (arrow) layers. Congested blood vessels (BV) and atretic follicles (AF) could be seen. (**E**) **PCOS + Adropin + Tirze Group** showing multiple corpora lutea (CL) mature Graafian follicles (GF) bordered with an outside thin theca cell layer (arrow) and an inner thick granulosa cell layer (star). Yet, few degenerated follicles (DF) and mild congested blood vessels (BV) are still present. [Magnification ×100 scale bar = 200 µm]. (**F**) A column graph showing the mean thickness of the granulosa and theca cell layer in the studied groups. ^a^ Significance versus CON group (*p* < 0.05), ^b^ Significance versus PCOS group (*p* < 0.05), ^c^ Significance versus PCOS + adropin group (*p* < 0.05) and ^d^ Significance versus PCOS + Tirze group (*p* < 0.05). n = 6 rats/each group.

The ovarian sections from the CON group showed normal histological architecture. The cortex indicated numerous follicles in various stages of development including mature Graafian follicles (Figure 5A). In the PCOS group, the ovarian sections depicted cystic follicles of varying sizes, (Figure 5B). Regarding the adropin-treated group, ovarian sections showed corpora lutea; however, few cystic follicles, degenerated follicles, and hemorrhage still existed (Figure 5C). Tirze administration resulted in fewer cystic follicles and more developing follicles. (Figure 5D). In contrast, in the adropin/Tirze combination group, the ovarian sections revealed a near-normal ovarian cortex. Nevertheless, a few degenerated follicles and mild congested blood vessels were present (Figure 5E). At a higher magnification, the CON group showed that growing follicles were enclosed by a thick granulosa layer and a thin theca cell layer (Figure 6A). In the PCOS group, the granulosa layer was thin and the theca cell layer was thick (Figure 6B). Regarding the adropin-treated group, ovarian sections showed moderately thick granulosa cell layers and theca cell layers (Figure 6C). In the Tirze administrated group, the theca cell layer was still thick (Figure 6D). In the adropin/Tirze combination group, the thickness of the granulosa and theca cell layers was near normal (Figure 6E).

#### 2.5.2. Statistical Evaluation of Morphometric Measures

Examination of the ovarian sections showed that the PCOS group’s mean number of developing follicles was much lower compared to the CON group. In contrast, it showed a highly significant rise with adropin and/or Tirze treatment relative to the PCOS group (Figure 5F). Comparing the PCOS group to the CON group, there was a significant decrease in the mean thickness of the granulose cell layer. By comparison, there was a significant rise in groups treated with adropin and/or Tirze as opposed to the PCOS group. Conversely, the CON group mean theca cell layer thickness was significantly lower than that of PCOS group. However, compared to PCOS groups, there was a highly significant drop in groups treated with adropin and/or Tirze treatment (Figure 6F).

**Figure 7:**
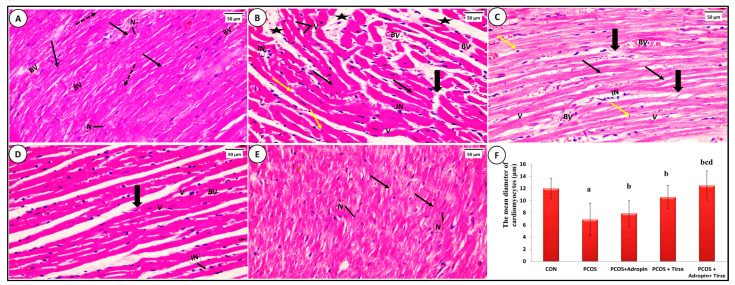
Sections of hearts stained with H&E. (**A**) **CON group** displaying longitudinally striated branching cardiomyocytes (arrows) with central oval vesicular nuclei (N) and acidophilic sarcoplasm as well as central oval vesicular nuclei (N). Blood capillaries (BV) and intercalated discs (dashed arrows) could be seen. (**B**) **The PCOS group** displaying disruption of some cardiac muscle fibers (thick arrows) that are widely separated from each other (stars). While some nuclei are pyknotic (yellow arrows), others are karyolitic (thin arrows). Many sarcoplasmic vacuolations (V). The blood vessels were dilated and congested (BV), and inflammatory cells (IN) were detected. (**C**) **PCOS + Adropin group** (thick arrow) demonstrating some atrophic and degraded fibers. In particular, the perivascular region exhibits cellular infiltration (IN) and congested blood vessels (BV). Observe the areas of localized sarcoplasmic vacuolations (V) and the karyolitic (thin arrow) and pyknotic (yellow arrow) nuclei. (**D**) **PCOS + Tirze group** demonstrating apparent normal cardiac muscles (thick arrows). Nevertheless, localized areas of sarcoplasmic vacuolations (V), slight congestion of blood vessels (BV), and a small number of mononuclear inflammatory cells (IN) are still observed. (**E**) **PCOS + Adropin + Tirze Group** displaying the maintenance of the heart muscles’ typical architecture, with almost regular cardiomyocytes (arrow) and an oval vesicular nucleus (N). [Magnification: 50 µm = ×400 scale bar]. (**F**) A column graph showing the mean diameter of cardiomyocytes in the studied groups. ^a^ Significance versus CON group (*p* < 0.05), ^b^ Significance versus PCOS group (*p* < 0.05), ^c^ Significance versus PCOS + adropin group (*p* < 0.05) and ^d^ Significance versus PCOS + Tirze group (*p* < 0.05). n = 6 rats/each group.

Examination of myocardial sections from the histological architecture of the CON group was typical, including branching and anastomosing cardiac muscle in various orientations. Muscle fibers showed acidophilic sarcoplasm and single oval centrally placed vesicular nuclei. Intercalated discs were typically seen running across the interval (Figure 7A). In contrast, the PCOS group showed marked disruption of cardiac muscle fibers, karyolytic or pyknotic nuclei, areas of sarcoplasmic vacuolations, dilated congested blood vessels, and interstitial inflammatory cells (Figure 7B). Sections from the adropin-treated group showed some atrophic and degenerated cardiac fibers with karyolitic and pyknotic nuclei and areas of focal sarcoplasmic vacuolations. There were noticeable congested blood vessels and cellular infiltration (Figure 7C). Tirze-treated rats sections showed seemingly normal heart muscles. Still, there were small sarcoplasmic vacuoles, somewhat congested blood capillaries, and a few mononuclear inflammatory cells (Figure 7D). On the other hand, the adropin/Tirze combination group demonstrated the return of the cardiac muscle fibers to their typical morphology, exhibiting almost regular cardiomyocytes with central vesicular nuclei (Figure 7E). When comparing the PCOS group’s mean cardiomyocyte diameter to the CON group, statistical analysis showed a significant reduction in this parameter, while groups treated with either Tirze or adropin and Tirze combination had a statistically significant increase in contrast to the PCOS group (Figure 7F).

### 2.6. Immunohistochemical Staining Study

#### NF-κB Immunohistochemical Staining

**Figure 8:**
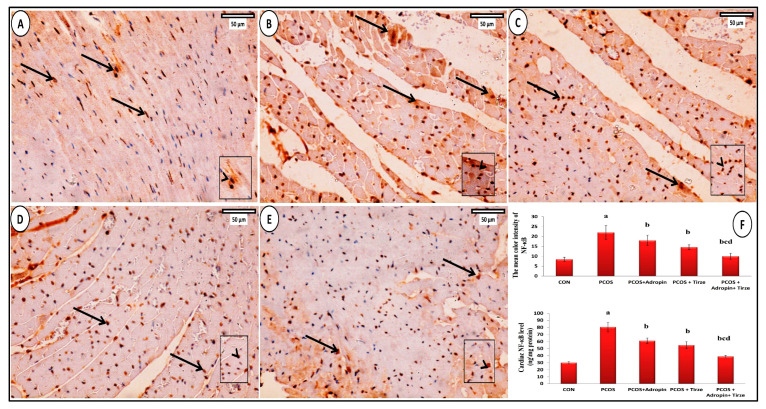
**NF-κB immunohistochemical stained myocardial sections from the studied groups.** (**A**) **CON Group** demonstrating a small number of cardiomyocytes with a moderately positive nuclear NF-κB immunoexpression (arrows). (**B**) **PCOS Group** showing that a large number of cardiomyocytes had nuclei with strong NF-κB nuclear immunoexpression (arrows). (**C**) **PCOS + Adropin Group:** has few cardiomyocytes with strong positive NF-κB immunoexpression (arrows). (**D**) **PCOS + Tirze Group:** had some cardiomyocytes with moderately positive NF-κB nuclear immunoexpression (arrows). (**E**) **PCOS + Adropin + Tirze group** depicting a small number of cardiomyocytes with moderately positive NF-κB nuclear immunoexpression (arrows). [Magnification: 50 µm = ×400 scale bar]. The intensity of the NF-κB nuclear immunoexpression is shown in higher magnification in the insert. (**F**) A column graph showing the mean color intensity of NF-κB immunostaining and its cardiac levels in the studied groups.^a^ Significance versus CON group (*p* < 0.05), ^b^ Significance versus PCOS group (*p* < 0.05), ^c^ Significance versus PCOS + adropin group (*p* < 0.05) and ^d^ Significance versus PCOS + Tirze group (*p* < 0.05). n = 6 rats/each group.

In the NF-κB-immunostained myocardial sections of the CON group, a small number of cardiomyocytes expressed moderate positive brown nuclear and/or perinuclear cytoplasmic immunoexpression for NF-κB (Figure 8A). In the PCOS group, numerous cardiomyocytes exhibited strong positive nuclear and/or perinuclear cytoplasmic immunoexpression for NF-κB (Figure 8B). Although the adropin-treated group presented a strong positive nuclear and/or perinuclear cytoplasmic immunoexpression for NF-κB only in a few cardiomyocytes (Figure 2C), the Tirze-treated group displayed a moderate positive nuclear immunoreaction for NF-κB in many cardiomyocytes (Figure 8D). In contrast, the adropin/Tirze combination induced a moderate positive nuclear immunoexpression for NF-κB in a few cardiomyocytes (Figure 8E).

Statistically, the PCOS group exhibited a substantial elevation in both the mean color intensity of NF-κB-positive immunoreaction and its cardiac levels relative to the CON group, which were notably decreased upon adropin and/or Tirze therapy (Figure 8F).

### 2.7. Bcl2 Immunohistochemical Staining

**Figure 9:**
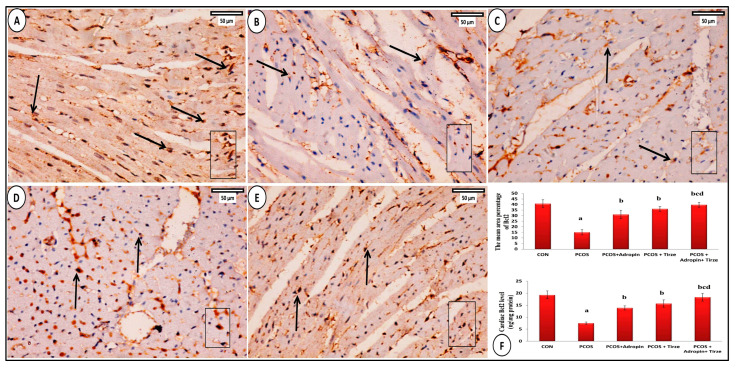
**Bcl2 immunohistochemical stained myocardial sections.** (**A**) **CON Group** displaying several cardiomyocytes with significant positive Bcl2 cytoplasmic immunoexpression (arrows). (**B**) **PCOS group** displaying cardiomyocytes with slightly positive Bcl2 cytoplasmic immunoexpression (arrows). (**C**) **PCOS + Adropin Group** depicting some cardiomyocytes with faint Bcl2 positive cytoplasmic immunoexpression (arrows). (**D**) **PCOS + Tirze group** demonstrating some cardiomyocytes with moderately Bcl2 positive cytoplasmic immunoexpression (arrows). (**E**) **PCOS + Adropin + Tirze Group** revealing several cardiomyocytes with moderately positive cytoplasmic Bcl2 immunoexpression (arrows). [Magnification: 50 µm = ×400 scale bar]. The intensity of the Bcl2 immunoexpression is shown at higher magnification in the insert. (**F**) A column graph showing the mean area percentage of Bcl2 immunostaining and its cardiac levels in the studied groups. ^a^ Significance versus CON group (*p* < 0.05), ^b^ Significance versus PCOS group (*p* < 0.05), ^c^ Significance versus PCOS + adropin group (*p* < 0.05) and ^d^ Significance versus PCOS + Tirze group (*p* < 0.05). n = 6 rats/each group.

The Bcl2 immunostained cardiac slices from the CON group showed that some cardiomyocytes had a significant positive cytoplasmic immunoexpression for Bcl2 (Figure 9A). Conversely, the PCOS group had a weak positive cytoplasmic immunoreaction for Bcl2 (Figure 9B). In the adropin-treated group, Bcl2 immunoexpression was weak in some cardiomyocytes (Figure 9C). The Tirze-treated group presented moderate positive cytoplasmic immunoreaction for Bcl2 in some cardiomyocytes (Figure 9D). Whereas, several cardiomyocytes in the adropin/Tirze combination group showed moderate positive Bcl2 immunoexpression (Figure 9E). In contrast to the CON group, the PCOS group scored a statistically significant drop in the mean area percentage of Bcl2 positive immunoexpression and its cardiac levels, which were notably increased following adropin and/or Tirze-treated treatment (Figure 9F).

### 2.8. Bax Immunohistochemical Staining

**Figure 10:**
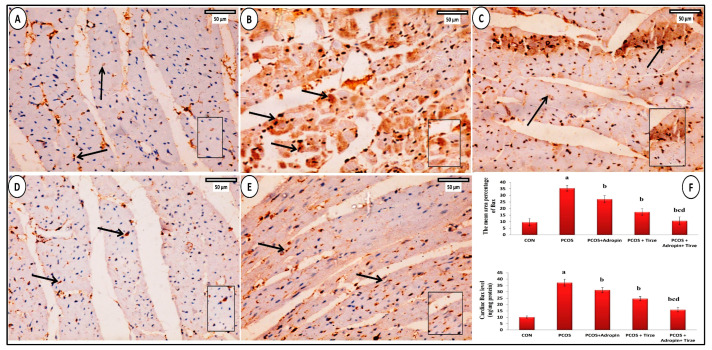
**Bax immunostaining.** (**A**) **CON group:** has a small number of cardiomyocytes with faint positive cytoplasmic Bax immunoexpression (arrows). (**B**) **PCOS Group** showing many cardiomyocytes with strongly positive Bax cytoplasmic immunoexpression (arrows). (**C**) **PCOS + Adropin Group** illustrating some cardiomyocytes with a somewhat strong Bax cytoplasmic immunoexpression (arrows). (**D**) **PCOS + Tirze Group** showing few cardiomyocytes with moderately positive Bax cytoplasmic immunoexpression (arrows). (**E**) **PCOS + Adropin + Tirze Group** displaying cardiomyocytes with weak positive Bax cytoplasmic immunoreaction (arrows). [Magnification: 50 µm = ×400 scale bar]. The intensity of the Bax immunoexpression is shown at higher magnification in the insert. (**F**) A column graph showing the mean area percentage of Bax immunostaining and its cardiac levels in the studied groups. ^a^ Significance versus CON group (*p* < 0.05), ^b^ Significance versus PCOS group (*p* < 0.05), ^c^ Significance versus PCOS + adropin group (*p* < 0.05) and ^d^ Significance versus PCOS + Tirze group (*p* < 0.05). n = 6 rats/each group.

The Bax immunostained myocardial sections from the CON group revealed a weakly positive Bax cytoplasmic immunoreaction in a small number of cardiomyocytes (Figure 10A). However, the PCOS group cytoplasmic immunoreaction for Bcl2 was strongly positive (Figure 10B). In adropin-treated rats, Bax immunoexpression was moderately positive in some cardiomyocytes (Figure 10C). The Tirze-treated group displayed a moderately positive cytoplasmic immunoreaction for Bax in cardiomyocytes (Figure 10D). However, few cardiomyocytes in the adropin/Tirze combination group showed weak positive Bax immunoexpression (Figure 10E). Comparing the PCOS group to the CON group, the mean area percentage of Bax-positive immunoexpression and its cardiac levels were statistically increased. In contrast, they exhibited a substantial decrement in adropin and/or Tirze-treated groups versus the PCOS group (Figure 10F).

### 2.9. Electron Microscope Examination

**Figure 11:**
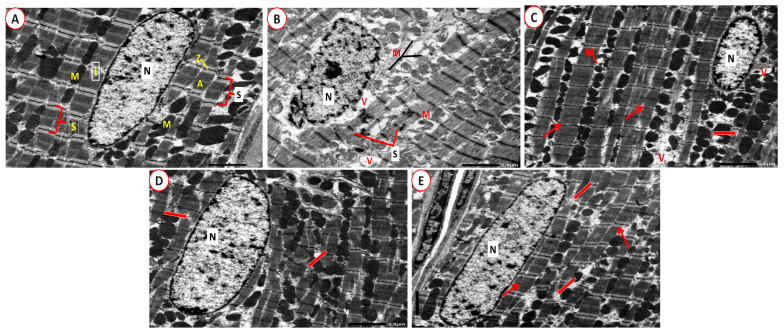
**Electron micrographs of rat myocardium.** (**A**) **CON Group** displaying a solitary euchromatic nucleus in a cardiomyocyte (N). Typical transverse myofibril striations consisting of dark (A) and light (I) bands divided by Z lines. Sarcomeres (S) are visible in the space between two consecutive Z-lines. Rows of mitochondria are located between the myofibrils and in the perinuclear region and, (M). (**B**) **PCOS Group** displaying a cardiomyocyte with a bizarre indented nucleus (N). Sarcomeres (S) lack their regular orientation. Swollen irregularly arranged mitochondria (M) as well as focal sarcoplasmic and perinuclear vacuolations (V) are noticeable. (**C**) **The PCOS + Adropin group** depicts a small nucleus in a cardiomyocyte (N) and regular transverse myofibril striations (thin arrows). However, focal lysis of cardiac myofibrils (thick arrow), few ruptured mitochondria (M), and focal sarcoplasmic vacuoles (V) are seen. (**D**) **PCOS + Tirze Group** demonstrates a euchromatic nucleus in cardiomyocyte (N) and focal lysis of cardiac myofibrils (thick arrows). (**E**) **PCOS + Adropin + Tirze Group** displays a cardiomyocyte with what seems to be regular transverse striations (thin arrows) and a sizable solitary euchromatic nucleus (N). Nevertheless, few myofibrils still depict focal areas of destruction (thick arrows). [Magnifications ×2500 scale bar =2 µm].

**Figure 12:**
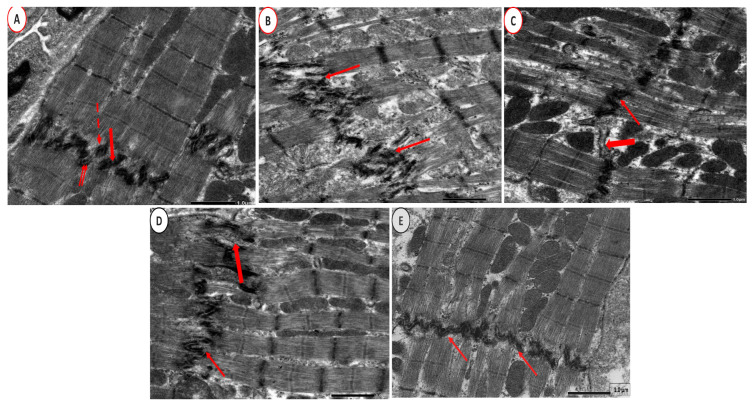
At a higher magnification. (**A**) **CON group** presentation step-like appearance of the three components of the intercalated discs, namely the desmosomes (thick arrow), fascia adherents (dashed arrow), and gap junction (double arrows), in between the myofibrils. (**B**) **PCOS Group** showing markedly interrupted and disfigured intercalated discs (arrows). (**C**) **PCOS + Adropin group** depicting step-like intercalated discs (arrows) with areas of dilatations (thick arrows). (**D**) **PCOS + Tirze Group** revealing step-like intercalated discs (arrows) with areas of interruption (thick arrows). (**E**) **PCOS + Adropin + Tirze Group** showing a normal step-like appearance of the three components of the intercalated discs (arrows). [Magnifications ×6000 scale bar = 1 µm].

Ultrastructural examination of CON group cardiac tissue sections revealed cardiomyocytes with euchromatic nuclei. The dark (A) and light (I) bands represented the regular arrangement of the cardiac transverse myofibril striations. Sarcomeres were located in between each pair of consecutive Z-lines. There were rows of mitochondria in the perinuclear region and in between the myofibrils (Figure 11A). Nevertheless, cardiomyocytes with irregular and indented nuclei were seen in the PCOS group. Furthermore, sarcomeres lack normal orientation, and swollen mitochondria and sarcoplasmic and perinuclear vacuolations were detected (Figure 11B). Adropin-treated group depicted a cardiomyocyte with a small euchromatic nucleus and what seem to be regular transverse myofibril striations. Nonetheless, there were a few ruptured mitochondria, there were localized sarcoplasmic vacuoles, and there was focal lysis of cardiac myofibrils (Figure 11C). Meanwhile, the Tirze-treated group exhibited a cardiomyocyte with a euchromatic nucleus and localized myofibril lysis (Figure 11D). In contrast, the adropin/Tirze combination group revealed a cardiomyocyte with a large single euchromatic nucleus and seemingly regular transverse striations. However, few myofibrils still depicted focal areas of destruction (Figure 11E). At higher magnification, the CON group had intercalated discs with a distinctive step-like appearance that joined the neighboring cardiomyocytes (Figure 12A). In the PCOS group, the intercalated discs were severely disturbed and distorted (Figure 12B). The adropin-treated group showed step-like intercalated discs with areas of dilatations that could be detected (Figure 12C) and step-like intercalated discs interrupted certain sites in the Tirze-treated group (Figure 12D). Despite this, the three intercalated disc components had a regular step-like look in the adropin/Tirze combination group (Figure 12E).

## 3. Discussion

The current research revealed, for the first time to the authors’ knowledge, the alleviating role of adropin and/or Tirze on PCOS-associated cardiometabolic abnormalities, with superiority of combination treatment, mostly mediated via modulating the cardiac Akt/GSK3β signaling pathway, which consequently ameliorated oxidative damage, abrogated the inflammatory processes, and modulated ER stress and associated apoptosis. In addition, they exhibited beneficial effects on biochemical, hormonal, and histological results, suggesting their therapeutic potential in regulating PCOS-associated reproductive and cardiometabolic disorders.

In line with Jahan et al. [19], our results elucidated that letrozole successfully persuaded PCOS in rats, manifested by a significant deterioration in glycemic control, both hormonal and lipid profiles, and histopathologic picture owing to its impacts on the hormonal homeostasis, increasing androgen levels, with a sequel of induction of IR.

Lower levels of adropin in the circulation were reported in women with PCOS, supporting the likelihood of its implication in the PCOS’ pathogenesis [20] Also, as adropin is crucial in regulating glucose and lipid metabolism, its role in the pathophysiology of PCOS and its related complications could not be ruled out.

Our findings revealed that adropin-treated PCOS rats exhibited a manifest decline in LH and testosterone serum levels, with notable improvement in the glycemic and lipid profiles, besides the histopathologic changes. In context with these results, earlier research documented adropin’s role in promoting insulin signaling pathways by phosphorylating Akt and increasing the expression of GLUT4 on the cell surface, in addition to modulating lipid metabolism via controlling the expression of hepatic lipogenic genes [21]. The evidence of the direct effects of adropin on PCOS is lacking, but these effects could be mediated indirectly through adropin’s substantial mitigation of the IR and glycemic control, which positively impacted the plasma LH and androgen levels [22]. Most recently, Rizk et al., in 2024, demonstrated a therapeutic effect of adropin on PCOS via controlling steroidogenesis, lipid profile, IR, and the gut microbiota inflammatory axis [23].

Recently, Tirze was hypothesized to be an innovative therapeutic approach for PCOS to address reproductive dysfunction, IR, and obesity [24]. Similarly, substantial improvements in both pancreatic beta-cell function and insulin sensitivity were noted with Tirze therapy [25]. The incorporation of both GIP and GLP-1 receptor stimulation demonstrated a more pronounced synergistic effect on insulin signaling pathways comparable to monotherapy [26]. Most recently, a meta-analysis study revealed that Tirze has favorable impacts on the whole lipid profile, particularly TG and VLDL-C [27]. In conformity, our results herein verified that Tirze treatment induced a substantial improvement in PCOS-induced alteration in both hormonal, glycemic, and lipid profiles, as well as alleviating the histopathologic changes.

Mounting evidence demonstrated that PCOS patients are at high risk of CVD mediated mostly by IR, in addition to hormonal and metabolic processes [28]. Adropin was suggested as an integral component of cardiometabolic diseases, where growing evidence posited that targeting adropin-related signaling pathways could benefit CVD, and further research was recommended to explicate the molecular pathways behind its possible therapeutic role [22]. Additionally, given the prominent role of GIP receptors in the ventricular myocardium and the reported Tirze-mediated cardiac protective effects in different models of type II diabetes and obesity [18,29,30], we hypothesized that Tirze and/or adropin might have a role in controlling cardiometabolic abnormalities in the induced PCOS rat model, which has not been demonstrated yet.

Cardiac impairment of insulin-induced glucose uptake was proposed as an early and consistent alteration in various models of myocardial IR, associated with impaired activation of the cardiac survival pathways mediated by Akt/GSK-3β, which could promote various cardiac pathogenic events [31,32].

GSK-3β phosphorylation by Akt activation results in its inactivation. The active dephosphorylated form of GSK3β enhances the activation with subsequent nuclear translocation of NF-κB p65, which consequently upregulates the expression of the NLRP3 inflammasome. This in turn promotes the proteolytic cleavage of pro-IL-1β and pro-IL-18 with a sequel of propagation of the inflammatory process and induction of apoptosis in heart tissues [33]. Thus, the modulation of the PI3K/AKT signaling pathway was proposed to be a candidate for the design of effective drugs for the treatment of heart disorders [34].

In line with this premise, the current work reported a substantial disruption in the cardiac AKT/GSK3β/NF-κB/NLRP3 axis of the PCOS rats that was reinforced by the disrupted histopathological picture. However, the administration of either adropin or Tirze effectively reversed such a disruption with better results for the combined intervention, as discussed in the result section.

These results were corroborated by prior studies demonstrating that adropin treatment regulated insulin signaling-related proteins in the heart including Akt and GSK-3β, thus supporting the therapeutic potential of adropin in treating cardiometabolic diseases [12,13]. Moreover, strong evidence documented the Tirze potentiality in counteracting abnormal IR responses through activation of PI3K/Akt with a sequel of suppression of GSk3-β in different tissues, including the heart [35,36,37]. Lastly, their anti-inflammatory potentials in different models of cardiac injury were attributed to their modulation of AKT/GSK-3β signaling and suppression of NLRP3 inflammasome activation [29,37,38,39,40].

NO-dependent oxidative stress and endothelial dysfunction are crucial factors contributing to cardiovascular pathophysiology in the PCOS setting, where generated reactive oxygen species cause uncoupling of endothelial nitric oxide synthase (eNOS), decreasing NO synthesis, which ultimately induces endothelial dysfunction and cardiac injury [41]. In accordance, the hearts of PCOS rats showed a marked increase in MDA and nitrite levels and a significant diminution of TAC levels compared to CON rats, suggesting an oxidative stress disruption, which was reversed by adropin and/or Tirze intervention.

The reported antioxidative properties of adropin among different tissues including the heart are attributed to its immune regulation function and activating nuclear factor erythroid 2-related factor 2 signaling, thus protecting mitochondrial function to alleviate oxidative stress and apoptosis [38,42,43]. Moreover, adropin could enhance the expression and activation of eNOS, increase NO production, and improve endothelial function [44].

Similarly, Tirze’s antioxidant criterion on tissue injury including the heart is well-documented [36,37,45]. This could be explained by activating Akt and its downstream signaling target forkhead box class O, inducing specific sets of nuclear genes, involving those combating oxidative stress [46]. Indeed, Tirze-specific impacts on NO production are lacking. Nevertheless, extensive studies documented the significance of GLP-1 receptor agonists in activating the eNOS pathway and increasing NO production [47].

The endoplasmic reticulum is a complex intracellular membranous network that modulates protein synthesis. Stress, like hyperglycemia or hypoxia, disrupts ER function, leading to misfolded protein accumulation and ER stress-related cardiomyocyte apoptosis. GRP78, a key ER-resident protein chaperone, activates the unfolded protein response when misfolded proteins accumulate, inhibiting protein translation and increasing ER protein-folding chaperones. Meanwhile, apoptotic cell death is prompted when the ER stress is prolonged, where CHOP is a key transcription factor in ER stress-mediated apoptosis [48].

Our results revealed a noteworthy upregulation of GRP78 and CHOP, a linker between ER stress and apoptosis, coupled with diminished immunoreactivity and levels of Bcl-2, and a rise in BAX and cleaved caspase-3 levels in the heart of PCOS rats. These effects were ameliorated upon adropin treatment, which could be mediated via its antioxidant and anti-inflammatory potentials demonstrated in this current research. Furthermore, its effect could be explained by modulating the cardiac AKT/GSK3β signaling, which was evidenced to play a role in controlling mitochondrial activity and inhibiting mitochondrial permeability pore opening [49]. To the best of the authors’ knowledge, this was the first study conducted to report the effect of adropin on ER stress in the heart of an induced PCOS rat model.

In addition, Tirze treatment notably reduced the overmentioned ER and apoptotic markers. This was in keeping with a most recent study reporting the possible role of Tirze in mitigating high glucose-induced apoptosis in cardiomyocytes [50]. Concerning the effect of Tirze on ER stress, information was scarce. Hassan et al., in 2024 [36], demonstrated that Tirze mitigated renal and neural injury-induced ER stress in rats. Treatment with a GLP-1/GIP dual agonist hampered ER stress and apoptosis in diabetic rats with cerebral ischemia-reperfusion injury as well [51]. Furthermore, it was indicated that GLP-1 could attenuate ER stress by triggering the GLP-1R/PI3K/Akt pathway, which ended in Bcl-2 release and consequently hindered apoptosis in cardiomyocytes [52].

## 4. Materials and Methods

### 4.1. Drugs and Reagents

Letrozole was provided by Sigma Aldrich Company (St. Louis, MO, USA). Adropin and Tirze were purchased from China Peptides Co., Ltd. (Shanghai, China), and Selleckchem (#P1206, Houston, TX, USA), respectively. Carboxymethyl cellulose (CMC) was obtained from El Gomhouria Pharmaceuticals Company for trading, chemicals, Cairo, Egypt. All other reagents were acquired from Sigma Chemical Co. (St. Louis, MO, USA). Letrozole was dissolved in 1% CMC solution. The Trize solution was created by mixing one milligram of Tirze powder with one milliliter of buffer (Tris-HCl pH8.0 + 0.02% PS80). The mixture was then put through a sterile Millipore filter, divided into aliquots, and kept at –80 °C.

### 4.2. Animals and Experimental Design

This research followed the National Institutes of Health guidelines for the use and care of laboratory animals (NIH Publications No. 85-23, revised 2011) and was permitted by the Research Ethics Committee, Tanta University, Faculty of Medicine, Egypt (Approval No. 36264PR149/3/23).

In this study, thirty adult non-pregnant female albino Wistar rats (8–10 weeks, weighing 200 ± 20 g) were housed in well-ventilated wire mesh cages (five rats per cage) under standard conditions at the temperature of 25 ± 5 °C with 50–60% relative humidity under a 12-h light/dark cycle and unrestricted access to food and water ad libitum during the experimental period. The chosen rats for this study were subjected to vaginal smears. Rats with at least 3 successive estrous cycles with the same estrous stage were allocated randomly into the later five equal groups after being acclimatized for one week, as follows:

- CON group: (1% CMC; 2 mL/kg/day, p.o.);
- PCOS group (letrozole; 1 mg/kg/day, p.o.) [53,54];
- PCOS + Adropin group (Adropin; 2.1 μg/kg/day, i.p.) [55];
- PCOS + Tirze group (Tirze; 1.35 mg/kg, s.c.) [35,56];
- PCOS + Adropin+ Tirze (received both adropin and Tirze concomitantly as previously mentioned).

Letrozole and CMC were administered for 21 days. Every day, during the last 12 days of letrozole administration, vaginal smears were collected, stained with Giemsa stain, and assessed microscopically to confirm induction of PCOS.

Then, adropin and Tirze in their known doses were given to the treated groups on the last day of letrozole administration for 14 successive days based on earlier studies [35,55,56], with some modifications based on a pilot study conducted by the authors.

### 4.3. Blood and Tissue Collection

After the experiment completion, the overnight fasted rats were anesthetized using sodium pentobarbital (60 mg/kg., i.p). After scarification, dry sterile centrifuge tubes were used to collect the blood samples from hearts. The blood samples were left for thirty minutes to coagulate at room temperature, then centrifuged for 20 min at 1000 g.

After that, the serum was isolated and kept at −80 °C for further biochemical assay. The ovaries were dissected out, defatted, and cleaned with ice-cold saline. The resultant supernatant of the right ovary homogenate was aliquoted and stored at −80 °C for investigating the tissue biochemical parameters. The left ovary was fixed in 10% neutral buffered formalin for histopathological examination.

Then, the heart was excised and cut into four pieces. Half of the left ventricle was preserved in 10% neutral buffered formalin for histopathological and immunohistochemical examinations. The other half was rapidly isolated and preserved in 2.5% phosphate-buffered glutaraldehyde for electron microscope examination. Both atria were homogenized, and the resultant supernatant was separated and kept at −80 °C for additional biochemical and immunoassay estimations. For molecular analysis, the right ventricles were kept at −80 °C. The total protein content of cardiac tissue was determined as previously defined by Lowry et al. [57].

### 4.4. Biochemical and Immunoassay Investigations

Fasting blood glucose level was assessed by the oxidase method using kits purchased from Biodiagnostic, Egypt. Serum levels of total cholesterol (TC), triglycerides (TG), and high-density lipoprotein (HDL-C) levels were analyzed using standard colorimetric kits supplied by Biodiagnostic, Egypt.

The malondialdehyde (MDA) level was detected in heart tissue homogenate by a colorimetric method depending on using an extinction coefficient of the MDA– thiobarbituric acid complex to calculate MDA concentration at 535 nm [58]. Cardiac total antioxidant capacity (TAC) was evaluated in a colorimetric manner defined by Koracevic et al. [59], while nitric oxide (NO) was evaluated calorimetrically at 540 nm by detecting its stable degradation products, nitrite, accumulation in the cardiac tissue homogenate using the Griess reagent [60].

Serum levels of insulin, luteinizing hormone (LH), and testosterone were scrutinized via the enzyme-linked immunosorbent assay (ELISA) technique using the commercial kits provided by Elabscience Biotechnology Inc, Houston, Texas, USA. Insulin resistance was estimated by calculating the homeostasis model assessment of IR (HOMA/IR) from this equation: HOMA-IR  =  insulin (μU/mL) × glucose (mg/dL)  ÷  405 [61] by multiplying fasting plasma glucose (mg/dL) by fasting plasma insulin (mIU/L) and then dividing the results by the constant 405.

Cardiac tissue levels of IL-1β, IL-18, pAKT, and pGSK3β were detected via ELISA kits provided by MyBioSource, Inc., San Diego, CA, USA, catalog # MBS825017; R&D Systems, Inc., Minneapolis, MN, USA, catalog # DY521-05; and MyBioSource, catalog # MBS1600201 and MBS730623, respectively.

Cardiac tissue levels of NF-κB, Bax, Bcl-2, GRP78, CHOP, and cleaved caspase-3 were scrutinized using ELISA kits catalog # MBS453975, MBS2512405, MBS2515143, MBS035991, MBS1608652, and SL1366Ra, respectively. For the cardiac CHOP, nuclear proteins were extracted using a nuclear extraction kit. Then, CHOP levels in the nuclear extract were ascertained by the corresponding ELISA kit. All ELISA procedures followed the manufacturer’s procedure and were perceived on a microplate reader at 450 nm ± 2 nm by the ELISA Reader (Star fax 2001).

### 4.5. Relative NLRP3, GRP78, and CHOP Gene Expression Assay by Quantitative Real-Time PCR

Cardiac tissue total RNA was extracted using the Direct-zol™ RNA MiniPrep (cat. no. R2051, ZYMO Research, Irvine, CA, USA) following the manufacturer’s directions, and then RNA concentration and quality were tested using NanoDrop 1000 (Thermo Fisher Scientific, Waltham, MA, USA). Next, the extracted RNA was converted to cDNA by reverse transcriptase using TOPscript™ RT DryMIX (dT18/dN6 plus) (cat. no. RT220, Enzynomics, Daejeon, Republic of Korea) according to the manufacturer’s guidelines. PCR reactions were accomplished by using TOPreal™ qPCR 2X PreMIX (SYBR Green with low ROX) (cat. no. RT500S, Enzynomics, Daejeon, Republic of Korea). Relative quantitation of mRNA expression was calculated according to the 2^−ΔΔCT^ method [62], in relation to the internal control gene glyceraldehyde-3-phosphate dehydrogenase (GAPDH) as a reference gene after validation. The primer sequences were rat NLRP3 (Gene Bank Accession No. NM_001191642.1: forward primer (F: 5′-GCTGCTCAGCTCTGACCTCT-3′) and reverse primer (R: 5′-AGGTGAGGCTGCAGTTGTCT-3′); rat GRP78 (Gene Bank Accession No. NM_013083.2): forward primer (F: 5′-GTTCTGCTTGATGTGTGTCC-3′) and reverse primer (R: 5′-TTTGGTCATTGGTGATGGTG-3′); rat CHOP (Gene Bank Accession No NM_001109986.1) forward primer (F: 5′-CTGCCTTTCACCTTGGAGAC-3′) and reverse primer (R: 5′-CGTTTCCTGGGGATGAGATA-3′); rat GAPDH (GenBank accession No. NM_017008.4): forward primer (F: 5′-CATGCCGCCTGGAGAAACCTGCCA-3′) and reverse primer (R: 5′-GGGCTCCCCAGGCCCCTC CTGT-3′). Non-template controls were included in each run to screen for any contamination. To monitor technical viabilities, 3 sample replicates were amplified blindly within each run to ensure an acceptable coefficient of variation. A melting curve analysis step was conducted to ensure the specificity of the PCR product.

### 4.6. Histological, Immunohistochemical, and Electron Microscopic Studies

For the histological study, specimens of the ovaries and left ventricle were preserved for a day in 10% buffered formalin, then embedded in hard paraffin blocks after being washed in xylol and put in pure soft paraffin. Hematoxylin–eosin (H&E) staining was utilized after the specimens were sliced into 3–5 μm thicknesses [63].

For the immunohistochemical study, specimens 5 µm thick that had been de-paraffinized, replenished, and cleaned in phosphate buffer saline were used for the immunohistochemical analysis. To inhibit the activity of endogenous peroxidase, it was later treated with 3% H2O2. Then, sections were manipulated with the primary antibodies; Nuclear factor kappa B (NF-κB) (p65) antibody (ab17742; Abcam, MA, USA), Anti-apoptotic protein Bcl2 (Thermo Fisher Scientific, Waltham, MA, USA, Cat. #PA5-27094); and rabbit polyclonal anti-Bax (ab53154; Abcam, MA, USA). To evaluate the response to immunostaining, slices were first preserved with the appropriate biotinylated secondary, the streptavidin peroxidase conjugate, and the diaminobenzidine (DAB) chromogen. Mayer’s hematoxylin was utilized as a counter-stain [64].

For transmission electron microscopic study, myocardial samples were fixed in glutaraldehyde at 4 °C, washed, dried, and treated with epoxy resin. The ultrathin fragments were cut. The slices were treated with uranyl acetate and lead citrate [65].

### 4.7. Morphometric Study

An image analysis computer system was used to analyze the following parameters. In each slide, five different non-overlapping fields were investigated at random.

1. Mean number of ovarian growing follicles (the primary, secondary, and antral follicles numbers were counted);
2. Mean thickness of the granulosa cell layer;
3. Mean thickness of the theca cell layer;
4. The mean diameter of cardiomyocytes;
5. Mean color intensity of NF-κB in immunostained sections;
6. Mean area %of Bcl2 in immunostained sections;
7. Mean area % of Bax in immunostained sections.

### 4.8. Statistical Analysis

Data analysis was conducted utilizing the Statistical Package of Social Science (SPSS) version 20 (SPSS Inc., Chicago, IL, USA). Mean ± standard deviation (SD) was used for representing the data, and the analysis was performed with the ANOVA test and the post-hoc Tukey’s test. A *p*-value of less than 0.05 was deemed statistically significant.

## 5. Conclusions

Our promising results provided compelling evidence for adropin and/or Tirze’s diverse modes of action in the PCOS model, with maximum beneficial effects reported for the combined intervention. This may be ascribed to their synergistic effects on activating the Akt and consequent disruption of the GSK3β/NF-κB/NLRP3-dependent signaling pathways, thereby alleviating oxidative damage, abrogating the inflammatory processes, and modulating ER stress and associated apoptosis. Thus, adropin and Tirze can be manipulated as a novel therapeutic candidate with the potential to tackle PCOS and its linked CVS complications.

## 6. Recommendations

Given the strong association between PCOS, metabolic disease, and dietary factors, future research should validate the potential of adropin/tirzepatide in dihydrotestosterone and diet-induced models. Such research could elucidate its role in addressing the broader PCOS phenotypes, particularly metabolic features, and boost the translational relevance of these findings to human PCOS. We also recommend incorporating techniques like Western blot, which could provide additional validation and mechanistic insights.

## Data Availability

The data that support the findings of this study are available from the corresponding author upon reasonable request.

## Abbreviations

AKT: protein kinase B; CHOP: C/EBP homologous protein; CMC: Carboxymethyl cellulose; ER: endoplasmic reticulum stress; GAPDH: glyceraldehyde-3-phosphate dehydrogenase; GIP: glucose-dependent insulinotropic polypeptide; GLP-1: glucagon-like peptide-1; *GRP78*: glucose-regulated protein 78; GSK-3β: glycogen synthase kinase-3 beta; HDL-C: high-density lipoprotein; HOMA/IR: homeostasis model assessment of IR; IL-18: interleukin 18; IL-1β: interleukin 1β; MDA: Malondialdehyde; NF-κB: nuclear factor kappa B; NLPR3: NOD-like receptor family pyrin domain containing 3; NO: Nitric oxide; PCOS: Polycystic ovarian syndrome; PI3K: phosphatidylinositol 3-kinase; TAC: total antioxidant capacity; TC: total cholesterol; TG: triglycerides; Tirze: tirzepatide.
